# Incommensurate structures and radiation damage in Rb_2_V_3_O_8_ and K_2_V_3_O_8_ mixed-valence vanadate fresnoites

**DOI:** 10.1107/S2052520623000999

**Published:** 2023-02-17

**Authors:** Andrzej Grzechnik, Vaclav Petříček, Dmitry Chernyshov, Charles McMonagle, Tobias Geise, Hend Shahed, Karen Friese

**Affiliations:** a Jülich Centre for Neutron Science (JCNS-4), Forschungszentrum Jülich GmbH, Jülich, 52425, Germany; b Institute of Crystallography, RWTH Aachen University, Aachen, 52066, Germany; c Institute of Physics of the Czech Academy of Sciences, Prague 8, 18221, Czech Republic; d Swiss–Norwegian Beamlines, European Synchrotron Radiation Facility, Grenoble, 38043, France; e Jülich Centre for Neutron Science (JCNS-2), Forschungszentrum Jülich GmbH, Jülich, 52425, Germany; SIMaP, France

**Keywords:** synchrotron radiation, single-crystal diffraction, incommensurate structures, radiation damage

## Abstract

The structures and phase transitions to incommensurate structures in Rb_2_V_3_O_8_ and K_2_V_3_O_8_ mixed-valence vanadate fresnoites are studied with synchrotron single-crystal diffraction at low temperatures and ambient pressure. Although mixed satellite reflections are absent, the modulated structure of K_2_V_3_O_8_ below 115 K is better described in (3 + 2)- than in (3 + 1)-dimensional space. The geometries of the VO_4_ and VO_5_ building units are rigid and it is mainly slight rotations of these polyhedra and small variation of the intermediate K—O distances that are modulated. Prolonged exposure to the high-brilliance synchrotron beam suppresses the incommensurate phase. The previously postulated phase transition to the incommensurate phase in Rb_2_V_3_O_8_ at 270 K was not observed. One of the reasons could be that the intense radiation also affects the modulation in this material. Strategies to collect and analyse single-crystal diffraction data measured with very intense synchrotron radiation using modern low-noise pixel area detectors are discussed.

## Abstract 2

The structures and phase transitions to the incommensurate structures in Rb_2_V_3_O_8_ and K_2_V_3_O_8_ mixed-valence vanadate fresnoites are studied with synchrotron single-crystal diffraction as a function of radiation dose at low temperatures and ambient pressure. Strategies to collect and analyse single-crystal diffraction data measured with very intense synchrotron radiation using modern low-noise pixel area detectors are discussed.

## 1. Introduction

**Figure 1:**
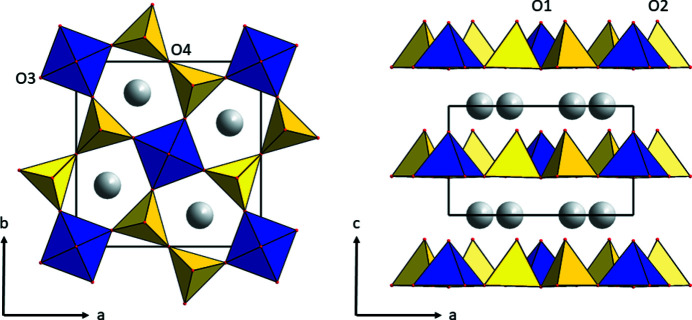
Crystal structures of the *A*_2_V_3_O_8_ vanadate fresnoites at room temperature.

Mixed-valence *A*_2_V_3_O_8_ vanadate fresnoites have a structure (*P*4*bm*, *Z* = 2) built up of layers with corner-sharing V^5+^O_4_ tetrahedra and V^4+^O_5_ square pyramids separated by the *A* ^+^ cations, where *A* ^+^ is K^+^, Rb^+^, Cs^+^ or (NH_4_)^+^ (Galy & Carpy, 1975 ▸; Theobald *et al.*, 1984 ▸; Ha-Eierdanz & Müller, 1992 ▸; Yeon *et al.*, 2013 ▸) (Fig. 1 ▸). Their magnetic and magneto-optical properties have been extensively investigated in the past (Liu & Greedan, 1995 ▸; Choi *et al.*, 2001 ▸, 2012 ▸; Sales *et al.*, 2002 ▸; Rai *et al.*, 2006 ▸; Lumsden *et al.*, 2006 ▸; Yeon *et al.*, 2013 ▸). K_2_V_3_O_8_ is a potential material for photocatalysis (Basu *et al.*, 2022 ▸) and batteries (Lu *et al.*, 2018 ▸; Jo *et al.*, 2019 ▸; Yang *et al.*, 2019 ▸; Li *et al.*, 2020 ▸, 2022 ▸),

The fresnoite Ba_2_TiSi_2_O_8_ mineral has an incommensurate (3 + 2)-*d* structure with modulation vectors **q**_1_ = 0.3020 (3)(***a*** *+ ***b*** *) and **q**_2_ = 0.3020 (3)(−***a*** *+ ***b*** *) and superspace group *P*4*bm*(α, α, ½)(−α, α, ½)0*gg* (Bindi *et al.*, 2006 ▸). The displacive modulations of the Ba^2+^ and O^2−^ ions lead to a variation in the coordination (eight-, nine- and tenfold) of the Ba^2+^ ions. The modulation of the TiO_5_ square pyramids and SiO_4_ tetrahedra is mainly rotational, where the former units stay essentially rigid while the pyrosilicate group also shows a significant change of geometry. On the other hand, the closely related compound Ba_2_TiGe_2_O_8_ has a (3 + 1)-*d* modulated structure with superspace group *Cmm*2(0, β, ½)*s*00, with a β component of the **q** vector of ∼0.635 (Höche *et al.*, 2003 ▸; Höche, 2005 ▸). The modulation can mainly be attributed to rotations of the TiO_5_ square pyramids and one of the pyrogermanate groups, while the second Ge_2_O_7_ unit is in addition deformed. This again leads to a variation of the coordination number of the barium ions between eightfold and tenfold. Based on these observations, Höche (2005 ▸) indicated that underbonding of the large cations (Ba^2+^ and Sr^2+^) is responsible for the displacive modulations in silicate and germanate fresnoites. In the average structures of the vanadium fresnoites K_2_V_3_O_8_ and Rb_2_V_3_O_8_, both K^+^ and Rb^+^ cations are overbonded (Höche, 2005 ▸).

Vibrational studies by Choi *et al.* (2001 ▸, 2012 ▸) revealed structural instabilities in K_2_V_3_O_8_ at about 110 K and 60 K. The former was argued to be due to a local distortion of the V^4+^O_5_ square pyramids. Later it was shown that K_2_V_3_O_8_ undergoes a structural phase transition at 115 K (Chakoumakos *et al.*, 2007 ▸; Takeda *et al.*, 2019 ▸). While there is an agreement in the literature on the fact that a modulated structure is formed below this temperature, there are discrepancies with respect to the modulation vector. Below 115 K, weak incommensurate satellite reflections were observed in electron diffraction patterns with the modulation vector **q**_1_ ∼ 0.12 ***c*** * (Höche, 2005 ▸). However, laboratory single-crystal X-ray data showed only weak satellites in the 〈110〉 projections at 90 K. These satellites were not analysed in detail and Höche (2005 ▸) only presented a refinement of the average structure (*P*4*bm*, *Z* = 2) at 90 K without taking satellite reflections into consideration in the analysis. The results of neutron powder diffraction and laboratory single-crystal X-ray diffraction on K_2_V_3_O_8_ suggested that weak superstructure reflections can be indexed with the commensurate wavevector 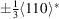 + ½ ***c*** * below 115 K (Chakoumakos *et al.*, 2007 ▸). The proposed possible space groups for the average structure are *P*4_2_ *bc* or *P*4*nc*. Based on single-crystal time-of-flight Laue neutron diffraction data, Wang & Petricek (2019 ▸) showed that K_2_V_3_O_8_ is incommensurately modulated and that the satellite reflections can be indexed with **q** vectors of ( 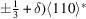 + ½ ***c*** *. They also discussed refinements in (3 + 2)-*d* tetragonal and (3 + 1)-*d* orthorhombic superspace groups.

Rb_2_V_3_O_8_, which is isostructural to K_2_V_3_O_8_ at ambient conditions, was found to undergo a phase transition to a modulated (3 + 1)-*d* structure at 270 K. Based on electron diffraction data, the wavevector was determined as **q**_1_ ∼ 0.16 ***c*** * and the superspace group was assumed to be *P*4*bm*(00γ) (Withers *et al.*, 2004 ▸; Höche, 2005 ▸). The modulation was supposed to arise from rotations and shifts of rigid V^5+^O_4_ and V^4+^O_5_ polyhedra. No incommensurate satellite reflections were detected with laboratory single-crystal X-ray diffraction in Rb_2_V_3_O_8_ (Withers *et al.*, 2004 ▸).

In this work, we are interested in the evolution of the average structures and the phase transitions to the incommensurate structures in Rb_2_V_3_O_8_ and K_2_V_3_O_8_ based on the synchrotron single-crystal diffraction at low temperatures and ambient pressure. Using the recently implemented cyclic refinement option in the program *Jana2020*, which is a modified version of *Jana2006* (Petříček *et al.*, 2014 ▸), we follow the temperature dependence of various unit cell and structural parameters of the average structures in the vicinity of the reported phase transitions at 270 K in Rb_2_V_3_O_8_ and at 115 K in K_2_V_3_O_8_ and examine details of the incommensurate phases. We also investigate the effect of the high-brilliance synchrotron beam on the crystal structures of both compounds.

## 2. Experimental

The synthesis of Rb_2_V_3_O_8_ and K_2_V_3_O_8_ crystals was as described by Lumsden *et al.* (2006 ▸). The preselection of the grown crystals for further synchrotron measurements was performed on laboratory X-ray diffractometers IPDS-II (Stoe) and Supernova (Rigaku Oxford Diffraction), both with Mo *K*α radiation. Each of the crystals in our study was about 50 µm × 40 µm × 20 µm in size. The IPDS-II data analysis with the *X-Area* (Stoe & Cie, 2011 ▸) program and refinements with the *Jana2020* (a modified version of *Jana2006*; Petříček *et al.*, 2014 ▸) program showed that the structures of Rb_2_V_3_O_8_ and K_2_V_3_O_8_ can be described in three-dimensional space (*P*4*bm*, *Z* = 2) at ambient temperature. The crystals contain only one enantiomorph, *i.e.* the refined volume fractions of the two individuals assuming inversion twinning were 1 and 0 within standard uncertainties.

Synchrotron single-crystal diffraction measurements (λ = 0.69127 Å) in the ranges 108–350 K for Rb_2_V_3_O_8_ and 88–298 K for K_2_V_3_O_8_ with a temperature step of 2 K at ambient pressure were performed on the BM01 station of Swiss–Norwegian Beamlines (SNBL) at the European Synchrotron Radiation Facility (Grenoble, France) (Dyadkin *et al.*, 2016 ▸). The crystals were mounted on glass pins and placed in a stream of nitro­gen from an Oxford Cryostream 700+ instrument. The data (a full rotation of 360°) were collected using a Pilatus 2M detector. After several test exposures, our data collection strategy was to measure frames with an angular step of 1° and an exposure time was 0.1 seconds per frame. Such a combination of an angular slicing and exposure time is a compromise that (i) keeps the detector in a linear response range even for strong reflections, (ii) uses rotation speed (10° s^−1^) that provides precise angular positioning, (iii) reduces the number of files to a reasonable and manageable number. Finer angular slicing is always better, but to stay with the same dose accumulation rate (same exposure per 0.1°) we would have to use the 0.1°/0.01 s strategy, which is still possible at BM01. Unfortunately, this implies 10 times more data are collected which is not very practical if the goal is to collect many datasets for mapping temperature or time evolution of the crystal structures. A discussion of data collection with pixel photon counting detectors at third-generation synchrotron sources was presented by Krause *et al.* (2020 ▸).

With an angular step of 1° and an exposure time of 0.1 seconds per frame, one data collection, *i.e.* one scan, took 36 s. The relatively high starting temperature (350 K) for the measurements on Rb_2_V_3_O_8_ was chosen so that several data points could be collected above the postulated phase transition at 270 K (Withers *et al.*, 2004 ▸). Using the same scan parameters, additional measurements to detect weak satellite reflections and to follow the evolution of the modulated structures as a function of the scan number (*i.e.* the exposure time or radiation dose) were performed at 100 K (λ = 0.69127 Å) and 250 K (λ = 0.60523 Å) for Rb_2_V_3_O_8_ on two different crystals with 20 and 40 scans, respectively. Similar measurements on one crystal of K_2_V_3_O_8_ at 100 K and 150 K were repeated 20 times (λ = 0.69127 Å). Additionally, they were repeated 360 times (λ = 0.60523 Å) on a second crystal of K_2_V_3_O_8_ at 100 K. The dose calculations were performed with *RADDOSE 3D* (Bury *et al.*, 2018 ▸) software. The average radiation dose per scan was 200 Gy on Rb_2_V_3_O_8_ and 160 Gy for K_2_V_3_O_8_. The corresponding frames from the separate scans were subsequently binned using the SNBL *ToolBox* (Dyadkin *et al.*, 2016 ▸) software. All the synchrotron data were analysed and processed with the program *CrysAlis* (Rigaku Oxford Diffraction, 2021 ▸). Solution and refinement of the structures were carried out with the program *Jana2020* (Petříček *et al.*, 2014 ▸). For this, we made use of the newly developed option of cyclic refinements for single crystal data, which was recently incorporated into *Jana2020* (Petříček *et al.*, 2014 ▸).

## 3. Results and discussion

**Figure 2:**
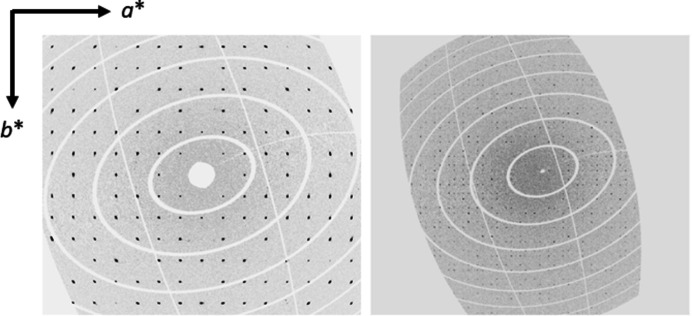
Reciprocal space sections *hk*0 (left) and *hk*0.5 (right) at 100 K.

A series of measurements on K_2_V_3_O_8_ was performed with single scans in the temperature range 88–298 K. Afterwards, the crystal was heated to 100 K and then to 150 K and 20 consecutive scans were collected at both temperatures. Analysis of reciprocal space in all the data shows that first-order satellite reflections can only be observed when the frames from 20 separate scans at 100 K are binned into one single data set (Fig. 2 ▸).

### 3.1. Temperature dependence of the average structure of K_2_V_3_O_8_

Considering solely the main reflections above and below the phase transition at 115 K, there is no indication for a change of space group *P*4*bm* of the average structure. Consequently, all the data collected with one scan between 88 K and 298 K were processed in space group *P*4*bm* using the cyclic refinement option in *Jana2020* (Petříček *et al.*, 2014 ▸). The starting data set for the cyclic refinement was the one measured at 88 K. It was refined first and, once a satisfactory agreement was achieved, the resulting structural parameters were then automatically passed on to the next higher temperature by the program. The next temperature point was then refined and this was subsequently repeated until the dataset at the highest temperature was reached. The refined parameters at all temperatures included anisotropic displacement parameters and an isotropic extinction correction, *G*_iso_. This new option in *Jana2020* also allows the direct plotting of the obtained structural parameters as a function of the corresponding variable, in this case the temperature. Since a trial refinement of the inversion twinning showed that the twin volume fractions for the enantiomorphs were 1 and 0 within standard deviation, no volume fractions were refined. The origin along the *c* direction was fixed to the position of the K atom (*z* = 0.5). Lowering symmetry to the non-isomorphic subgroups *P*4, *Cmm*2 and *Pba*2, and introducing additional twinning due to the loss of rotational symmetry elements in the refinements of the data below 115 K did not improve the result for the average structure, despite the increased number of free parameters.

**Figure 3:**
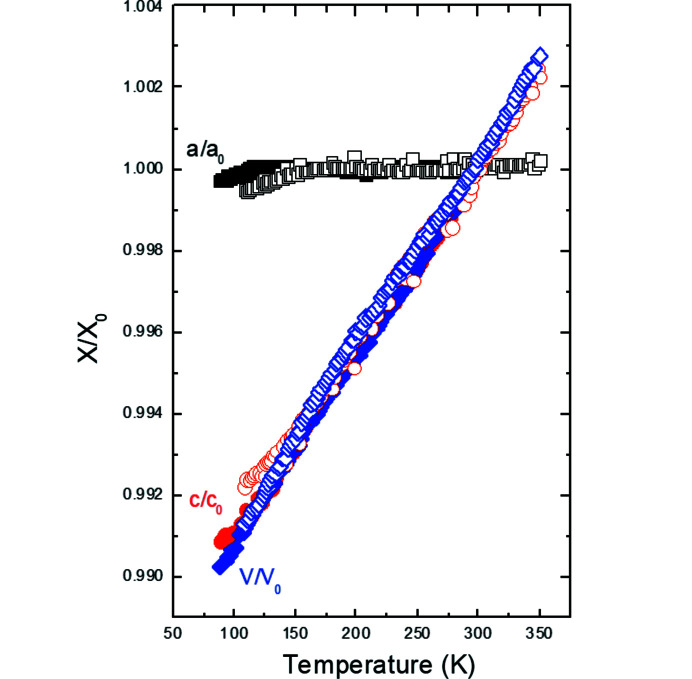
Normalized unit-cell parameters and unit-cell volumes to the values at 298 K as a function of temperature. The open and solid symbols are for Rb_2_V_3_O_8_ and K_2_V_3_O_8_, respectively.

**Figure 4:**
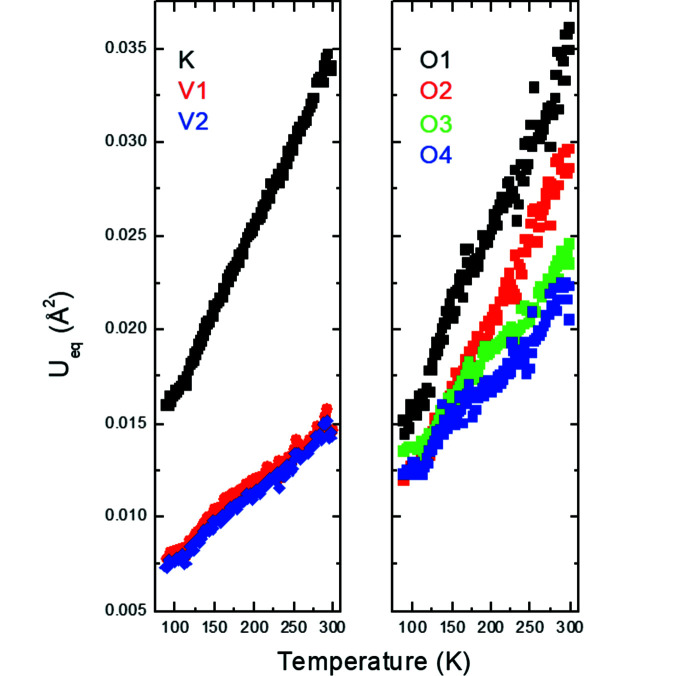
*U*_eq_ atomic displacement parameters as a function of temperature in K_2_V_3_O_8_. The standard uncertainties are smaller than the size of the symbols.

The temperature dependence of the normalized unit-cell parameters and unit-cell volumes of the average structure, *P*4*bm*, in K_2_V_3_O_8_ is shown in Fig. 3 ▸. Down to about 115 K, the *a* unit-cell parameter is essentially constant and the volume contraction is entirely due to the change in the *c* unit-cell parameter in K_2_V_3_O_8_. At lower temperatures, the *a* unit-cell parameter contracts while the temperature dependence of the *c* unit-cell parameter changes its slope. The volume contraction is altogether linear in the entire temperature range 88–298 K, *V*(*T*) = 412.77 (1) + 0.01902 (5)*T. The anomalies in the evolution of the *a* and *c* unit-cell parameters indicate the phase transition at 115 K in K_2_V_3_O_8_ reported earlier (Choi *et al.*, 2001 ▸, 2012 ▸; Höche, 2005 ▸; Chakoumakos *et al.*, 2007 ▸; Takeda *et al.*, 2019 ▸). This phase transition is also reflected in the slope changes of the isotropic displacement parameters for all the atoms in the average structure (Fig. 4 ▸).

**Figure 5:**
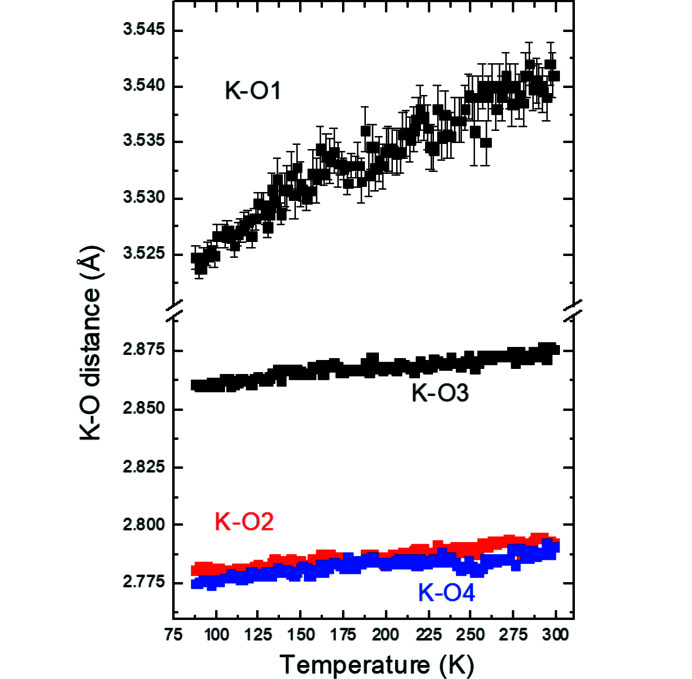
K–O interatomic distances as a function of temperature in K_2_V_3_O_8_. The standard uncertainties are drawn when larger than the size of the symbols.

**Figure 6:**
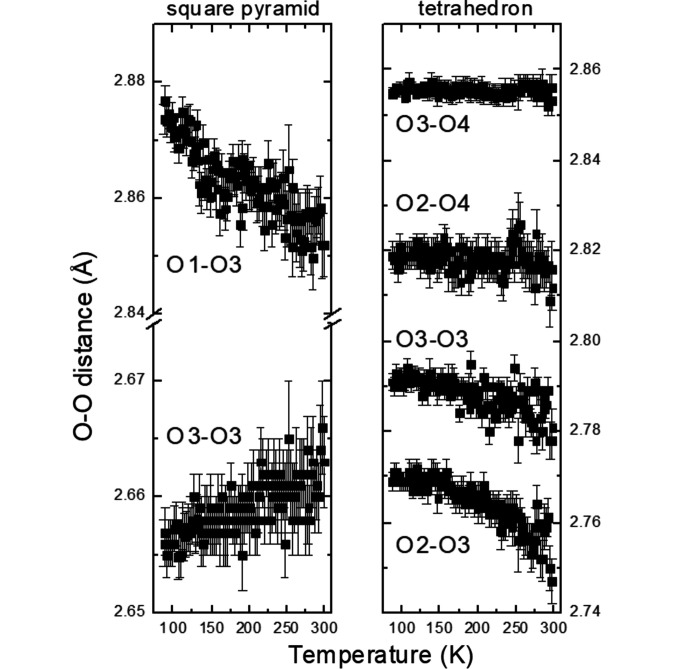
O–O interatomic distances as a function of temperature in K_2_V_3_O_8_.

**Figure 7:**
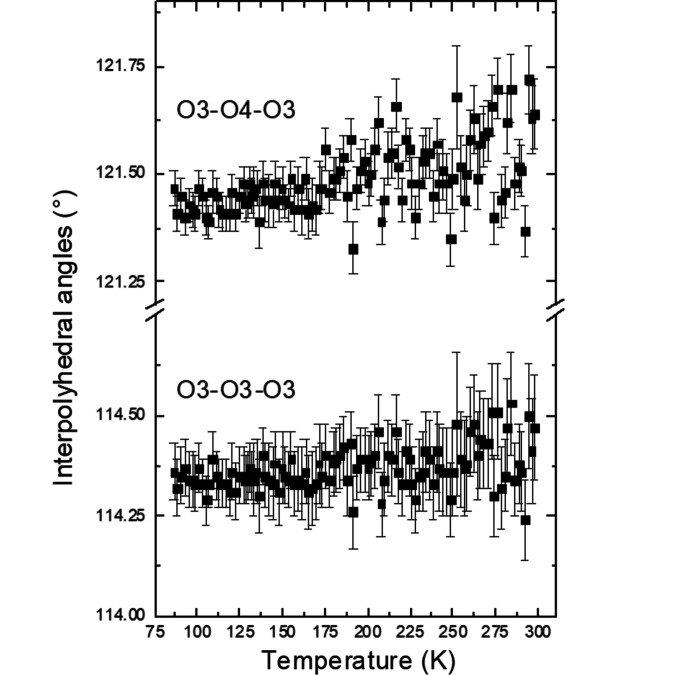
Interpolyhedral angles as a function of temperature in K_2_V_3_O_8_.

It is striking that there are no obvious anomalies in various geometrical parameters such as interatomic distances and angles at the phase transition at 115 K in K_2_V_3_O_8_ (Figs. 5 ▸–7 ▸ ▸, additional Figs. S1–S4 are in the supporting information). The unit-cell contraction along the *c* axis in the entire temperature range studied here is mainly due to the shortening of the K–O distances between the V_3_O_8_ slabs (Fig. 5 ▸). This observation is supported by slightly increasing volumes of the VO_4_ tetrahedra and VO_5_ square pyramids upon cooling calculated using the program *ToposPro* (Blatov *et al.*, 2014 ▸). The distances V1—O1 and O1—O3 (the O1 and O3 atoms being at the apex and the base of the VO_5_ polyhedron, respectively) increase upon cooling indicating that the square pyramid becomes elongated (Fig. 6 ▸). On the other hand, the V—O and O—O distortions of the VO_4_ tetrahedra (as defined by Baur, 1974 ▸) decrease. The O3–O4–O3 and O3–O3–O3 angles between two tetrahedra and between the tetrahedra and square pyramids, respectively, are essentially constant (Fig. 7 ▸).

### 3.2. Incommensurate structure of K_2_V_3_O_8_

No satellite reflections are seen in reciprocal space sections of our data with 20 binned scans recorded at 150 K. The very weak first-order satellite reflections, which appear in the *hk*0.5 section of reciprocal space at 100 K, can be described with two vectors **q**_1_ = (α, α, 0.5) and **q**_2_ (−α, α, 0.5), where α ∼ 0.3 (Fig. 2 ▸). No higher-order satellites or satellite reflections due to combination of the **q**_1_ and **q**_2_ vectors are visible in the corresponding sections of reciprocal space perpendicular to [001]. We also do not detect any satellites with the modulation vector **q** ∼ 0.12 ***c*** * reported earlier by Höche (2005 ▸). These observations allow two different interpretations of the structure of K_2_V_3_O_8_ below 115 K: (i) the structure is (3 + 2)-*d* or (ii) the structure is (3 + 1)-*d* and the observed diffraction pattern can be explained on the basis of additional twinning due to a 90° rotation around ***c*** *. The fact that in the latter case all satellites can be indexed with one single vector **q**_1_ = (0, β, 0.5) in combination with twinning would also imply that the overall symmetry is no longer tetragonal.

We first performed a refinement in (3 + 2)-*d* space. Starting from the space group *P*4*bm* of the average structure and the two **q** vectors, **q**_1_ = (0.313, 0.313, ½) and **q**_2_ = (−0.313, 0.313, ½), all different translational parts for *x*4 and *x*5 of two superspace group symmetry operators were considered and tested (altogether 16 possibilities). The combination leading to superspace group *P*4*bm*(*αα*½)(−αα½)0*gg* led to the best refinement result. Two modulation waves both for the positions and anisotropic displacement parameters of all the atoms were considered.^1^ The refinement converged to about *R*(all) = 2.07%, 1.31% and 7.17% for all reflections, main reflections, and first-order satellites, respectively (Table 1 ▸). The superspace group *P*4*bm*(*αα*½)(−αα½)0*gg* is analogous to the one observed in the natural fresnoite Ba_2_TiSi_2_O_8_ (Bindi *et al.*, 2006 ▸), but with different **q** vectors.

The second model in (3 + 1)-*d* space implies that the modulation happens along one direction only. Therefore, orthorhombic symmetry is more likely for the (3 + 1)-*d* model. It was derived from the (3 + 2)-*d* one by reducing symmetry to orthorhombic (*C*-centred cell with *a*_ortho_ = *a*_tetra_ − *b*_tetra_, *b*_ortho_ = *a*_tetra_ + *b*_tetra_, *c*_ortho_ = *c*_tetra_) and changing the number of modulation waves from two to one. The resulting superspace group for the (3 + 1)-*d* model is then *Cmm*2(0β½)*s*00 with one modulation vector **q**_1_ = (0, 0.626, ½). Two twin domains originating from the loss of the fourfold axis were introduced. The twin volume fractions refined to 0.469 (2):0.531 (2). Final refinement values for the four-dimensional model are *R*(all) = 2.40%, 1.39% and 8.48% for all, main and satellite reflections (Table S1 in the supporting information). The superspace group and **q** vector of this model correspond to those of Ba_2_TiGe_2_O_8_ fresnoite (Höche *et al.*, 2003 ▸; Höche, 2005 ▸).

As the detection of mixed satellites would allow to uniquely identify the (3 + 2)-*d* model as the correct one, we collected a larger number of scans (360) on a second crystal and then binned them together (the crystal was in the beam for one scan at room temperature to check its quality). Even in these data, no mixed satellites could be observed in K_2_V_3_O_8_. Therefore, an unambiguous decision about the correct model for the modulated structure of K_2_V_3_O_8_ is not straightforward. As agreement factors for the (3 + 2)-*d* model are slightly better than for the (3 + 1)-*d* one and as the number of parameters in the refinement is significantly lower (96 parameters as compared to 144, respectively), the (3 + 2)-*d* model is more likely. Also, a comparison of the standard uncertainties of the atomic coordinates and atomic displacement parameters shows, that the errors on the parameters are on average 3–4 times smaller in the (3 + 2)-*d* model then in the (3 + 1)-*d* model.

The diffraction image simulations based on the refined (3 + 2)-*d* model using harmonic waves in principle do generate extremely weak mixed satellite reflections. It explains why these satellites could not be detected in our data, in which the first-order satellites are already very weak. If the modulations were stronger, the satellite formation would indeed exist. This, of course, is not a direct evidence that the structure is (3 + 2)-*d*, but it confirms that such a model does not contradict what was observed in this study. Consequently, the following discussion will mainly focus on the tetragonal (3 + 2)-*d* model (Table S1 and Figs. S5–S7 corresponding to the (3 + 1)-*d* model are provided in the supporting information).

**Figure 8:**
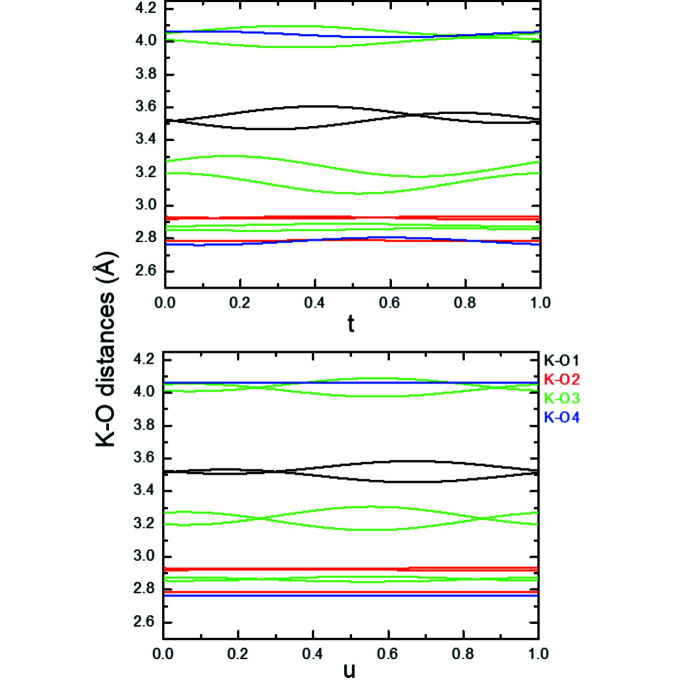
K–O distances below 4.25 Å in the (3 + 2)-*d* structure of K_2_V_3_O_8_ at 100 K as a function of the internal coordinate *t* (*u* = 0.0) (top) and *u* (*t* = 0.0) (bottom).

In both models of the modulated structure of K_2_V_3_O_8_ at 100 K the geometries of both VO_4_ and VO_5_ building units are basically rigid. The V—O distances and O—V—O angles in both polyhedra are constant within standard uncertainties as a function of the internal coordinates *t* and *u* (see Bindi *et al.*, 2006 ▸). In addition, K—O distances below 3 Å are also only very weakly affected by the modulation and it is mainly the intermediate K—O distances (3.0–3.75 Å) that show a variation with the internal parameter(s) (Fig. 8 ▸, see also Fig. S5). In contrast to the earlier investigated modulated structures of fresnoites, where the bond valence sums (BVS) of the *A* cations are significantly affected by the modulation, the bond valence sum of the K atom in K_2_V_3_O_8_ is hardly affected by the varying distances and oscillates in the range BVS_K_ = 0.947 (1) − 0.949 (1) v.u. throughout the modulated structure.

**Figure 9:**
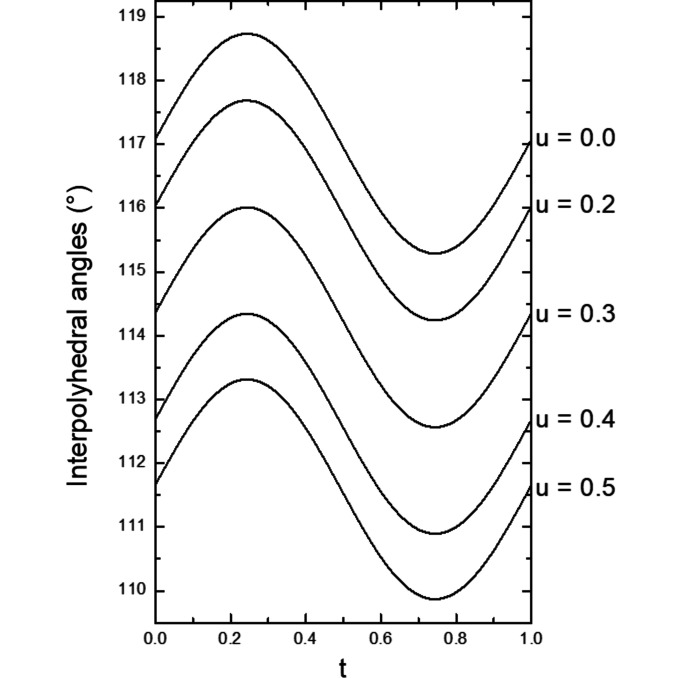
O3–O3–O3 interpolyhedral angles in the (3 + 2)-*d* model as a function of the internal coordinate *t* for different values of the internal coordinate *u*.

The main influence of the modulation is on the interpolyhedral angles of the tetrahedra of the pyrovanadate group, O3—O3—O3, and on the angles between the tetrahedra and the square pyramids, O3—O4—O3 (Figs. 9 ▸ and 10 ▸, see also Figs. S6 and S7). The variation of these angles leads to a slightly varying shape of the five membered rings surrounding the K atoms.

### 3.3. Influence of the radiation dose on the stability of the incommensurate structure of K_2_V_3_O_8_

**Figure 11:**
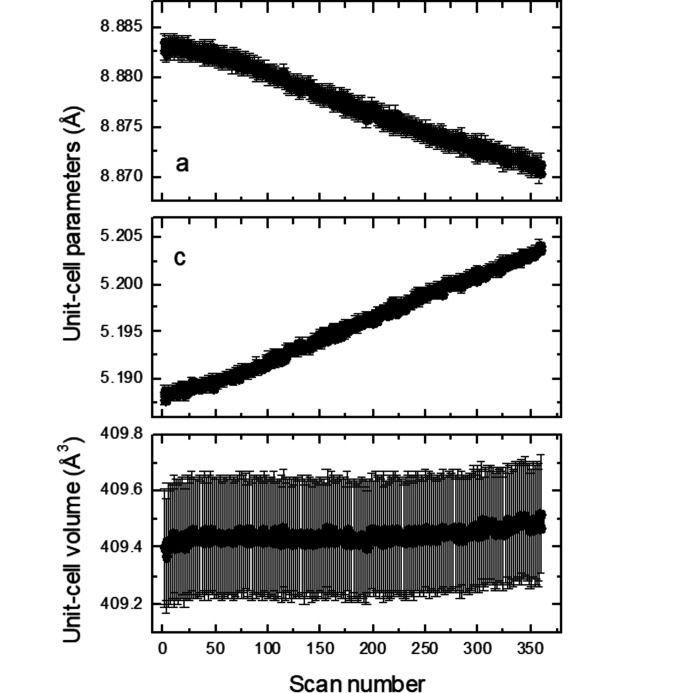
Unit-cell parameters and unit-cell volumes as a function of the scan number in K_2_V_3_O_8_ at 100 K. Standard uncertainties of all the parameters are plotted in the figure.

**Figure 12:**
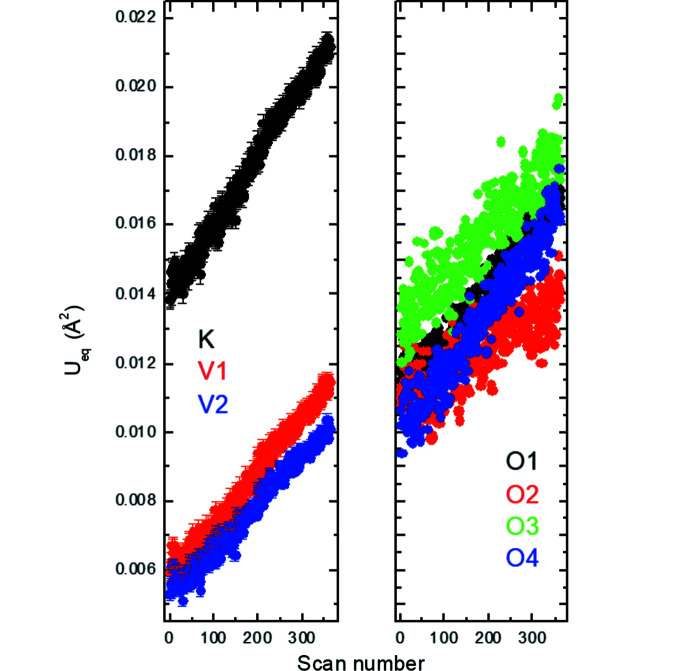
*U*_eq_ atomic displacement parameters as a function of the scan number in K_2_V_3_O_8_ at 100 K. Standard uncertainties for the oxygen atoms are not plotted for clarity.

**Figure 13:**
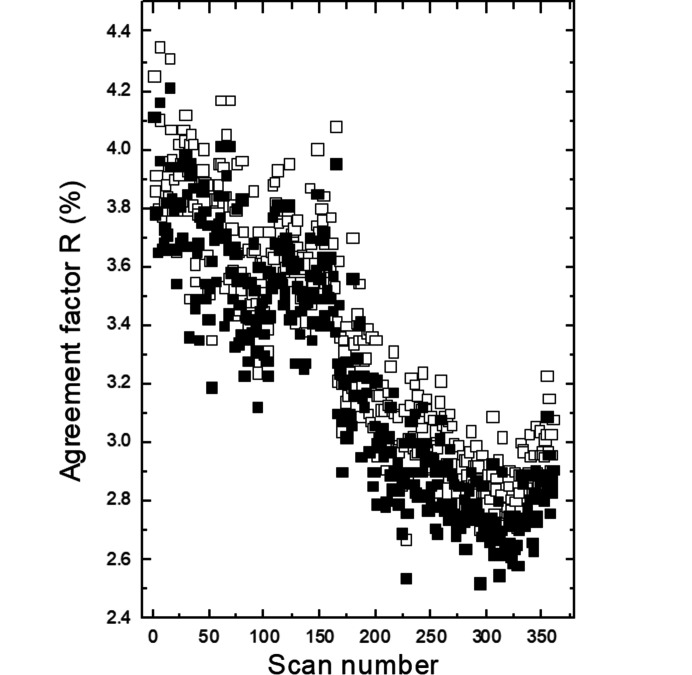
Agreement factors *R*_obs_ (full symbols) and *R*_all_ (open symbols) as a function of the scan number in K_2_V_3_O_8_ at 100 K.

A close inspection of the whole series of 360 scans, which were measured on the second crystal, reveals that the first-order satellites were only visible when the initial 40 to 50 scans at 100 K were binned together. Binning frames corresponding to higher scan numbers did not show the presence of satellites and did therefore not contain any information about the modulation. Binning the first 40–50 scans in consecutive groups of 10 or 20 showed that the magnitude α of the vectors **q**_1_ = (α, α, 0.5) and **q**_2_ = (−α, α, 0.5) in the (3 + 2)-*d* model or of the vector **q** = (0, β, 0.5) in the (3 + 1)-*d* model are constant within the estimated standard deviations, *i.e.* α ≃ 0.313 (2) or β ≃ 0.626 (2). The satellites just became weaker and subsequently completely disappeared for higher scan numbers or, in other words, for increased exposure time or radiation dose. To elucidate the effect of the radiation dose on the average structure of K_2_V_3_O_8_ below 100 K, we refined all the 360 scans with the cyclic option in *Jana2020* (Petříček *et al.*, 2014 ▸). It is worth noting that the *a* unit-cell parameter is constant up to about scan No. 50, above which it decreases (Fig. 11 ▸). This coincides with the disappearance of the satellites. On the other hand, the *c* unit-cell parameter increases, while the unit-cell volume is constant. The response of the crystal structure of K_2_V_3_O_8_ to the high-brilliance synchrotron beam is also visible in the increase of the isotropic displacement parameters for all the atoms (Fig. 12 ▸). Both agreement factors *R*_obs_ and *R*_all_ decrease with the radiation dose (Fig. 13 ▸).

### 3.4. Temperature and radiation dose dependence of the structure of Rb_2_V_3_O_8_

**Figure 14:**
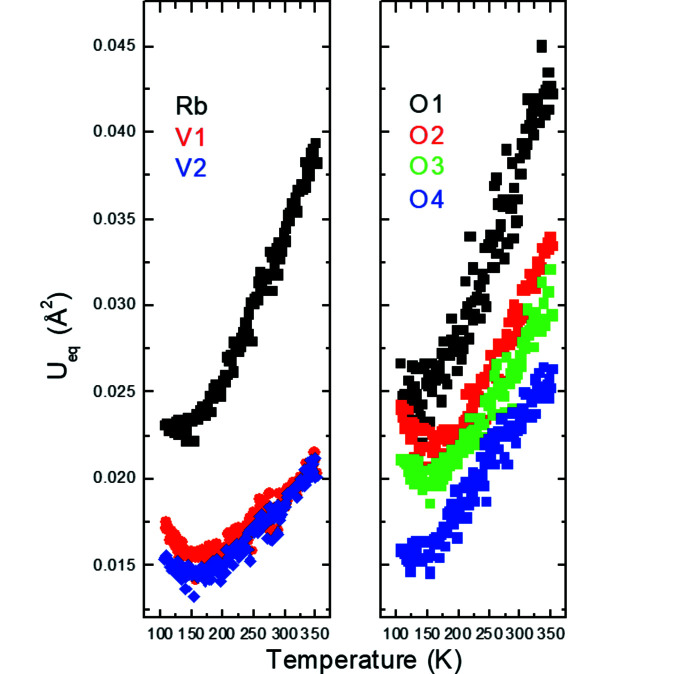
*U*_eq_ atomic displacement parameters as a function of temperature in Rb_2_V_3_O_8_. Standard uncertainties are smaller than the size of the symbols.

A similar approach as the one described above for K_2_V_3_O_8_ was followed to determine the temperature-dependent behaviour of Rb_2_V_3_O_8_. The data with one scan were recorded on cooling in the temperature range 108–350 K and then refined in a cyclic way using the program *Jana2020* (Petříček *et al.*, 2014 ▸). Based on the analysis of the reciprocal space at all temperatures, there is no evidence for a change of space group *P*4*bm* of the average structure. It is striking that the anomalies in the *a* and *c* unit-cell parameters occur in Rb_2_V_3_O_8_ at about 180 K (Fig. 3 ▸). The bulk thermal contraction also changes its slope at this temperature indicating higher thermal contraction below 180 K. The volume contraction above and below 180 K is *V*(*T*) = 438.32 (1) + 0.0197 (1)*T and *V*(*T*) = 437.61 (2) + 0.0238 (2)*T, respectively. We do not see any anomaly in the temperature evolution of the unit-cell parameters, unit-cell volumes and geometrical parameters at 270 K, which would correspond to the occurrence of the previously reported modulated structure (Withers *et al.*, 2004 ▸; Höche, 2005 ▸). On the other hand, the isotropic displacement parameters become constant or increase below about 180 K (Fig. 14 ▸). This would possibly indicate the presence of a transition to a phase with a different symmetry than *P*4*bm*. However, neither magnetic measurements by Liu & Greedan (1995 ▸) nor thermal analysis by Withers *et al.* (2004 ▸) showed any evidence for a phase transition at this temperature.

To check whether there is indeed a new phase of Rb_2_V_3_O_8_ below 180 K, we collected 20 consecutive scans at 100 K on the same crystal at the end of the series of measurements in the range 108–350 K. It turned out that in the binned data set at this temperature there are no additional weak reflections violating the extinction rules for space group *P*4*bm* of the average structure. We also do not see any weak features that could possibly be interpreted as satellites or superstructure reflections.

**Figure 15:**
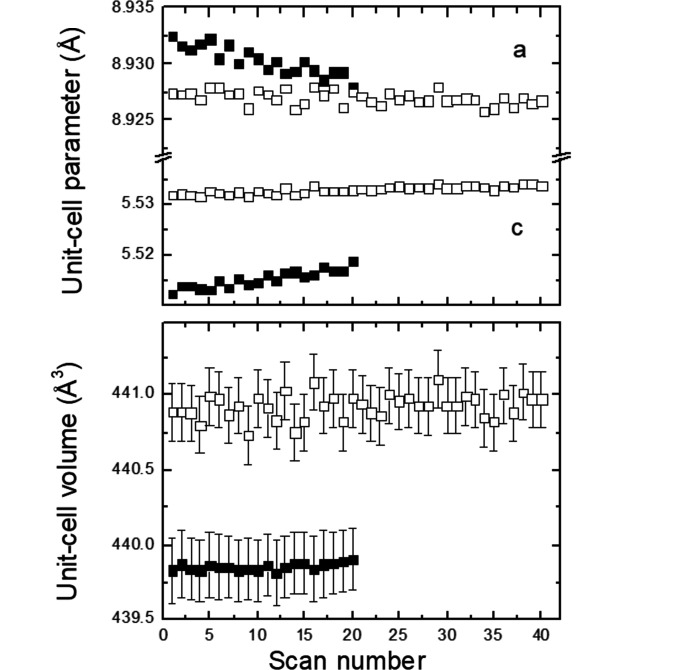
Unit-cell parameters and unit-cell volumes as a function of the scan number in Rb_2_V_3_O_8_ at 100 K (full symbols) and 250 K (open symbols). Standard uncertainties are shown when larger than the size of the symbols.

**Figure 16:**
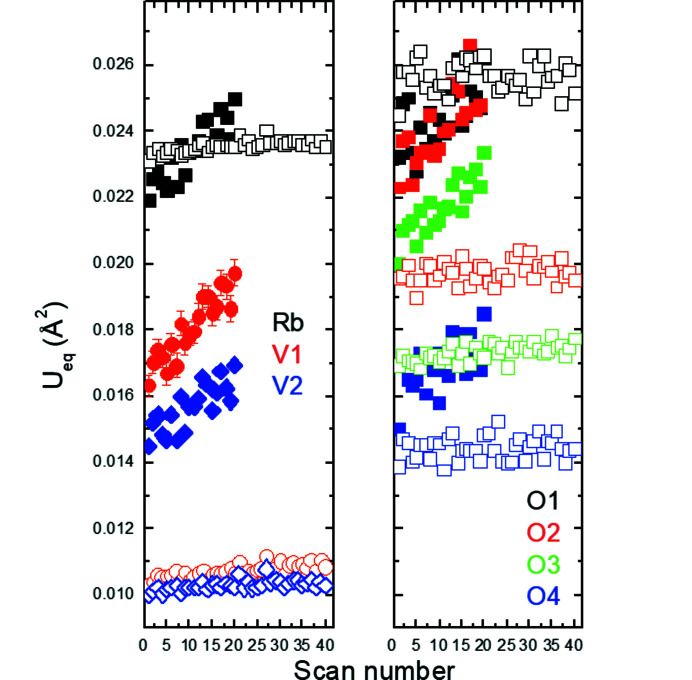
*U*_eq_ atomic displacement parameters as a function of the scan number in Rb_2_V_3_O_8_ at 100 K (full symbols) and 250 K (open symbols). Standard uncertainties for the cations are shown when larger than the size of the symbols. Standard uncertainties for the oxygen atoms are not plotted for clarity.

A cyclic refinement of the 20 scans collected on the first crystal of Rb_2_V_3_O_8_ at 100 K with the program *Jana2020* (Petříček *et al.*, 2014 ▸) as a function of the scan number (*i.e.* the exposure time or radiation dose) revealed that the *a* and *c* unit-cell parameters slightly decrease and increase, respectively (Fig. 15 ▸). In addition, the isotropic displacement parameters for all atoms monotonically increase with the constant unit-cell volume (Fig. 16 ▸).

An additional data set with 40 scans was collected on a second crystal of Rb_2_V_3_O_8_ at 250 K, *i.e.* below the phase transition to the incommensurate phase reported by Withers *et al.* (2004 ▸), without any prior long exposure to the synchrotron radiation. The crystal was in the beam for one scan at room temperature to check its quality. No satellites are seen in the combined data sets from either first 20 or all 40 binned scans at 250 K. Both the *a* unit-cell parameter and unit-cell volume remain essentially constant while the *c* unit-cell parameter slightly increases as a function of the scan number (Fig. 15 ▸). The response of the crystal structure to the prolonged synchrotron beam exposure is also reflected in the increase of the isotropic displacement parameters for all the cations (Fig. 16 ▸). These trends at both 100 K and 250 K are in principle analogous to those observed upon cooling below 180 K and to those in K_2_V_3_O_8_ at 100 K upon long exposure to the synchrotron radiation beam. Unlike in K_2_V_3_O_8_ at 100 K (Fig. 13 ▸), the agreement factors *R*_obs_ and *R*_all_ for the data on Rb_2_V_3_O_8_ with limited number of scans at 100 K and 250 K (20 and 40, respectively) are essentially constant.

## 4. Conclusions

In the temperature range in which the commensurate fresnoite structures of K_2_V_3_O_8_ and Rb_2_V_3_O_8_ (*P*4*bm*, *Z* = 2) are stable, the *a* unit-cell parameter is constant and the thermal volume contraction is solely due to the change in the *c* unit-cell parameter. Such a behaviour is very similar to that of (NH_4_)_2_V_3_O_8_ and Cs_2_V_3_O_8_ upon compression at room temperature (Grzechnik *et al.*, 2011 ▸, 2016 ▸). Up to the pressure-induced phase transitions to the centrosymmetric structures *P*4/*mbm*, which are observed in these two vanadate fresnoites, the *a* unit-cell parameters are also nearly constant and it is mainly the contraction perpendicular to the stacking of the V_3_O_8_ slabs that accounts for the bulk compressibility.

Due to the absence of mixed satellite reflections it is not possible to unambiguously decide whether the modulated structure of K_2_V_3_O_8_ below 115 K is better described in (3 + 2)- or (3 + 1)-dimensional space. However, the slightly better agreement factors and the smaller number of parameters hint towards the (3 + 2)-*d* model as the correct choice. The geometries of the VO_4_ and VO_5_ building units stay constant as a function of the modulation and it is mainly slight rotations of these polyhedra (reflected in varying interpolyhedral O—O—O angles) that lead to a small variation of the intermediate K—O distances. In contrast to other fresnoites, the effect of the modulation on the bond valence sums of the slightly ‘underbonded’ K^+^ are minimal.

Since no bulk thermal expansion is observed in K_2_V_3_O_8_ and Rb_2_V_3_O_8_ when they are in the high-brilliance synchrotron beam for an extended time, we interpret our observations on their behaviour to be caused from the exposure to radiation. Any effect from heating the material by the beam would otherwise lead to the bulk expansion of the lattice. Our results imply that the detection of weak satellites in incommensurate phases (*e.g.* the mixed satellites in K_2_V_3_O_8_ below 115 K) is difficult as the prolonged exposure to a high-brilliance synchrotron beam could lead to the disappearance of subtle modulations. The onset of the damage in Rb_2_V_3_O_8_ could already be seen at a temperature as high as 250 K. The postulated phase transition to the incommensurate phase, which is supposed to occur at 270 K (Withers *et al.*, 2004 ▸), is not observed in our synchrotron data. The reason could be either that the intense radiation suppresses the modulation or that the electron beam, to which the sample was exposed in the study by Withers *et al.* (2004 ▸), shows an interaction with the structure that leads to the development of a modulated structure.

Radiation damage manifests as a structural disorder in the crystal, that suppresses scattering at high angles and can heat the crystal and/or modify the equilibrium between various polymorphic modifications. Notable examples in the field of small molecule crystallography were presented by Christensen *et al.* (2019 ▸), Lawrence Bright *et al.* (2021 ▸), Bogdanov *et al.* (2021 ▸), Chernyshov *et al.* (2022 ▸). However, none of these examples deals with modulated structures.

The newly developed cyclic refinement option in *Jana2020* (Petříček *et al.*, 2014 ▸) permits a convenient treatment of many subsequent data sets collected on one single crystal under similar conditions. We could show that this option is not only suitable for temperature-dependent measurements but also for the study of the effects due to the radiation damage.

We finally conclude with a note on synchrotron data collection strategies suitable to measure coexisting strong and weak diffraction features. Bright synchrotron light and modern low-noise pixel area detectors make diffraction experiments very fast, but there is a limitation on the linear detector response of the dynamic range. One strategy is to collect data with different attenuations of the incoming beam and then merge the datasets, taking the strong intensities from the attenuated data and weak intensities from the un-attenuated measurement. Another strategy, the one applied here, is to perform the same data collection many times with an attenuated beam. These data can then be summed partially or totally into a single dataset with improved statistics. Such binning of the data is a technique perfectly suited to the zero-readout noise pixel area detectors, such as the Pilatus 2M used in our case. Individual datasets can be analysed separately to see the effect of the radiation dose as each dataset accounts for a specific dose accumulation proportional to the exposure time. This collection strategy gives greater flexibility for data analysis over more traditional strategies and allows the optimum quality data to be extracted from each crystal.

## Supplementary Material

Crystal structure: contains datablock(s) global, I-5D, I-4D. DOI: 10.1107/S2052520623000999/dq5056sup1.cif

Structure factors: contains datablock(s) I-5D. DOI: 10.1107/S2052520623000999/dq5056I-5Dsup2.hkl

Structure factors: contains datablock(s) I-4D. DOI: 10.1107/S2052520623000999/dq5056I-4Dsup3.hkl

Supporting information file. DOI: 10.1107/S2052520623000999/dq5056sup4.pdf

z034DL74QTM

CCDC references: 2239698, 2240704

## Figures and Tables

**Figure 10:**
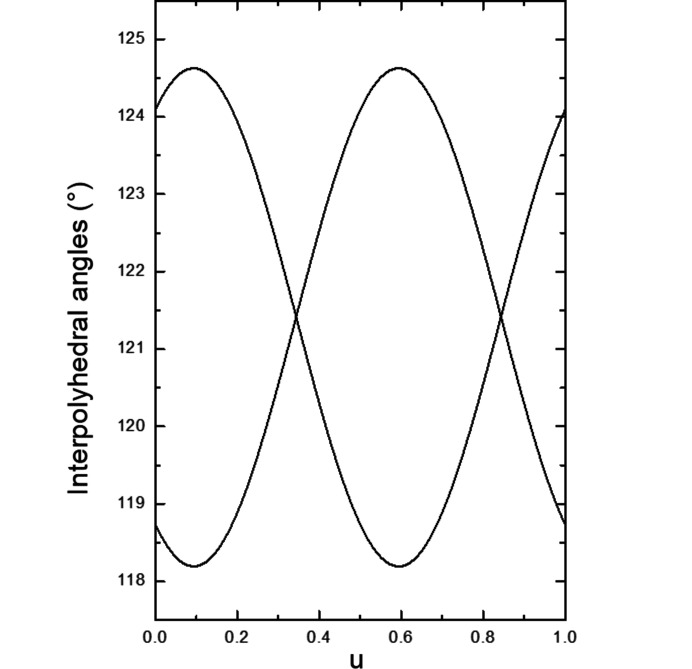
O3–O4–O3 interpolyhedral angles in the (3 + 2)-*d* model as a function of the internal coordinate *u*. The modulation of the angles does not depend on the internal coordinate *t*.

**Table 1:** Experimental data for the measurement on the (3 + 2)-*d* structure of K_2_V_3_O_8_ at 100 K (λ = 0.69127 Å)

Superspace group	*P*4*bm*(*αα*½)(−αα½)0*gg*
*a* (Å)	8.942 (2)
*c* (Å)	5.224 (2)
*V* (Å^3^)	417.6 (3)
*Z*	2
*D*_calc_ (g cm^−3^)	2.855
*G*_iso_	0.208 (8)
	
Modulation vector **q**_1_	0.313 (2), 0.313 (2), 0.499 (1)
Modulation vector **q**_2_	−0.313 (2), 0.313 (2), 0.499 (1)
	
Data collection	
No. measured reflections	8245
No. main reflections	1463
No. satellite reflections ± (1,0)	3794
No. observed reflections (all, main, satellites)†	1240, 446, 794
*R*_int_(obs, all)	1.68, 1.70
	
Refinement‡	
*R*_obs, all_ (all reflections)	1.56, 2.07
*wR*_obs,all_ (all reflections)	1.93, 2.00
*R*_obs,all_ (main reflections)	1.31, 1.31
*wR*_obs,all_ (main reflections)	1.69, 1.69
*R*_obs,all_ [satellite reflections ± (1,0)]	4.00, 7.17
*wR*_obs,all_ [satellite reflections ± (1,0)]	4.10, 4.58
No. of parameters	96

† Criterion for observed reflections is |*F*_obs_| > 3σ.

‡ All agreement factors are given in %, weighing scheme 1/[σ^2^(*F*_obs_) + (0.01 *F*_obs_)^2^].

## Footnotes

1 When the Fourier amplitudes of the modulation function of the displacement parameters were smaller than three times the standard deviation, the corresponding parameters were set to 0.0 in both models. This did not lead to a significant increase of the overall agreement factors.
