# Supplementary material for: Incommensurate structures and radiation damage in Rb2V3O8 and K2V3O8 mixed-valence vanadate fresnoites
Source: Acta Crystallogr B Struct Sci Cryst Eng Mater. 2023 Feb 17;79(Pt 2):104–13. doi: 10.1107/S2052520623000999 (PMC10088480; doi:10.1107/S2052520623000999)
Supplement: Supplementary file 4 [file b-79-00104-sup4.pdf]

STRUCTURAL SCIENCE
CRYSTAL ENGINEERING
MATERIALS

Volume 79 (2023)

Supporting information for article:

Incommensurate structures and radiation damage in Rb2V3O8 and K2V3O8 mixed-valence vanadate fresnoites

Andrzej Grzechnik, Vaclav Petricek, Dmitry Chernyshov, Charles McMonagle, Tobias Geise, Hend Shahed and Karen Friese

Table S1 Experimental data for the measurement on the (3+1)-*d* structure of $K_2V_3O_8$ at 100 K (● = 0.69127 Å).

Superspace group	*Cmm*2(0*β*1/2)*s*00
a (Å)	12.6452(9)
b (Å)	12.6452(9)
c (Å)	5.2235(5)
V ($Å^3$)	835.2
Z	4
D_c (g cm^{-3})	2.855
Twin volume (1,2)	0.469(2), 0.531(2)
G_{iso}	0.214(6)
Modulation vector **q**	0, 0.626, 0.5
Data collection	
No. measured refl.	8351
No. main refl.	1575
No. satellite refl. ± (1,0)	6776
Range of *hkl*	$-16 < h < 15$
	$-16 < k < 16$
	$-6 < l < 6$
	$-1 < m < 1$
No. observed refl. (all, main, satellites)[a]	1929, 762, 1167
R_{int}(obs/all)	1.58/1.61
Refinement [b]	
$R_{obs/all}$ (all refl.)	1.58/2.40
$wR_{obs/all}$ (all refl.)	1.98/2.09
$R_{obs/all}$ (main refl.)	1.34/1.39
$wR_{obs/all}$ (main refl.)	1.80/1.83
$R_{obs/all}$ (satellite refl. ± (1,0))	4.09/8.48
$wR_{obs/all}$ (satellite refl. ± (1,0))	4.49/5.31
No. parameters	144

[a] Criterion for observed reflections is $|F_{obs}| > 3$⬧

[b] All agreement factors are given in %, weighing scheme 1/[⬧^{2}(F_{obs}) + (0.01 F_{obs})^2]

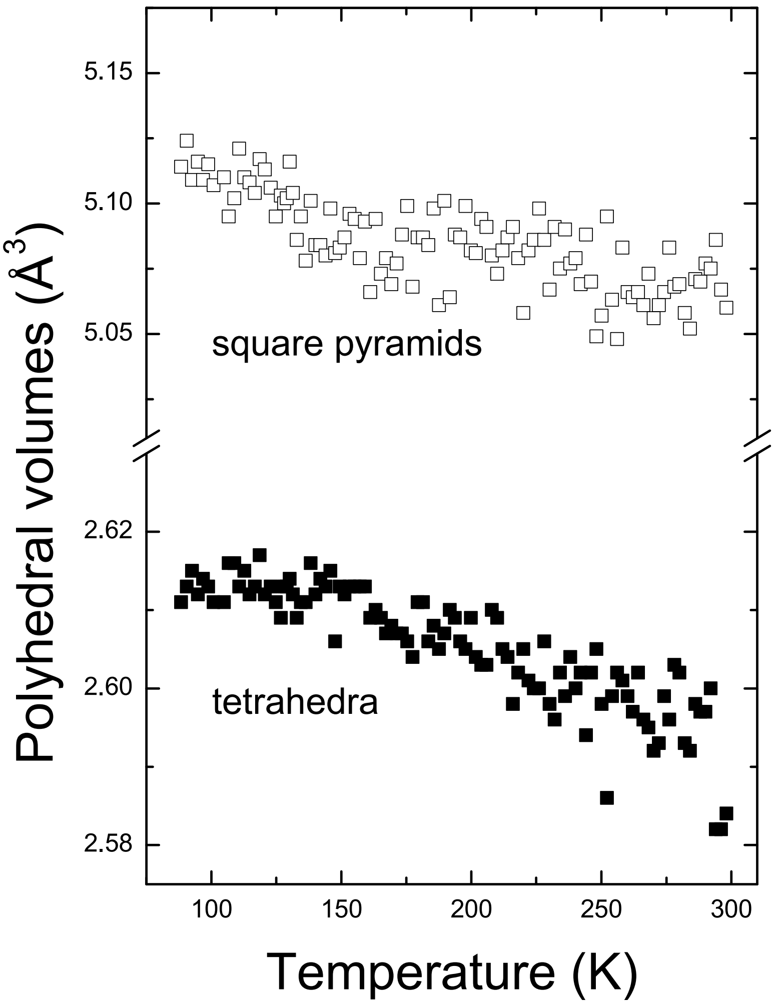

Figure S1 Volume of the tetrahedra and square pyramids as a function of temperature in $K_2V_3O_8$.

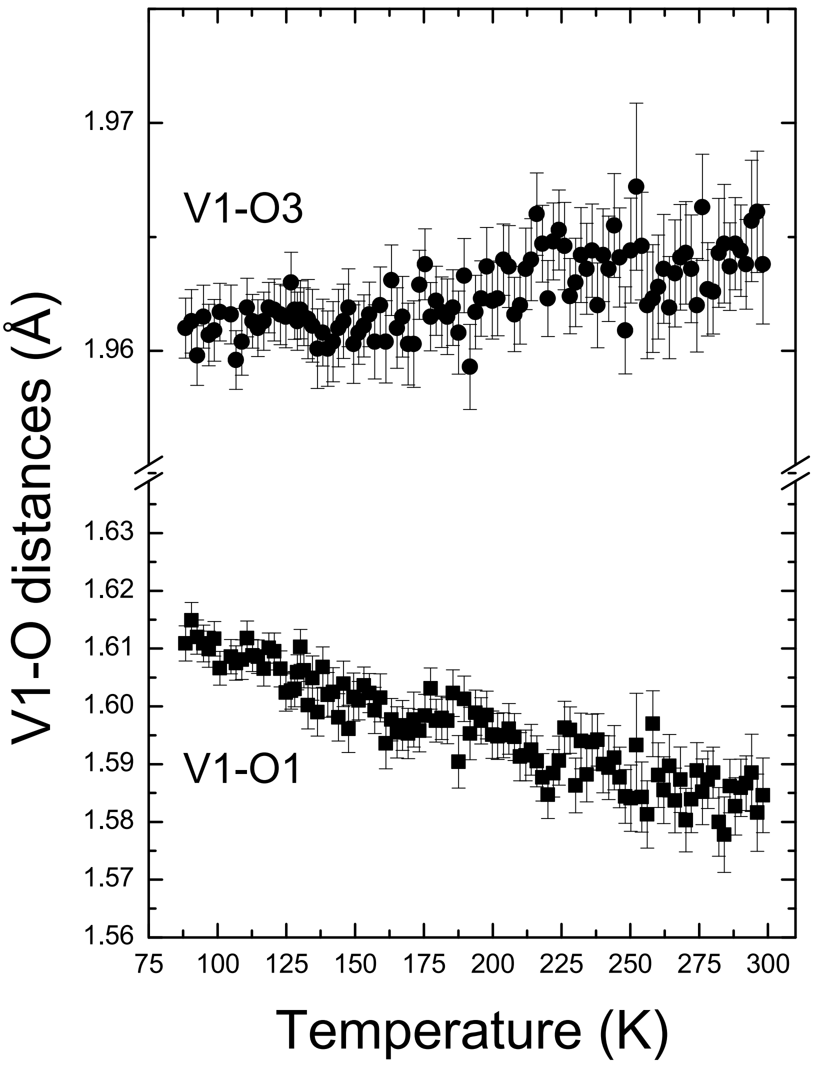

Figure S2 V1-O interatomic distances in the VO_5 square pyramid as a function of temperature in $K_2V_3O_8$.

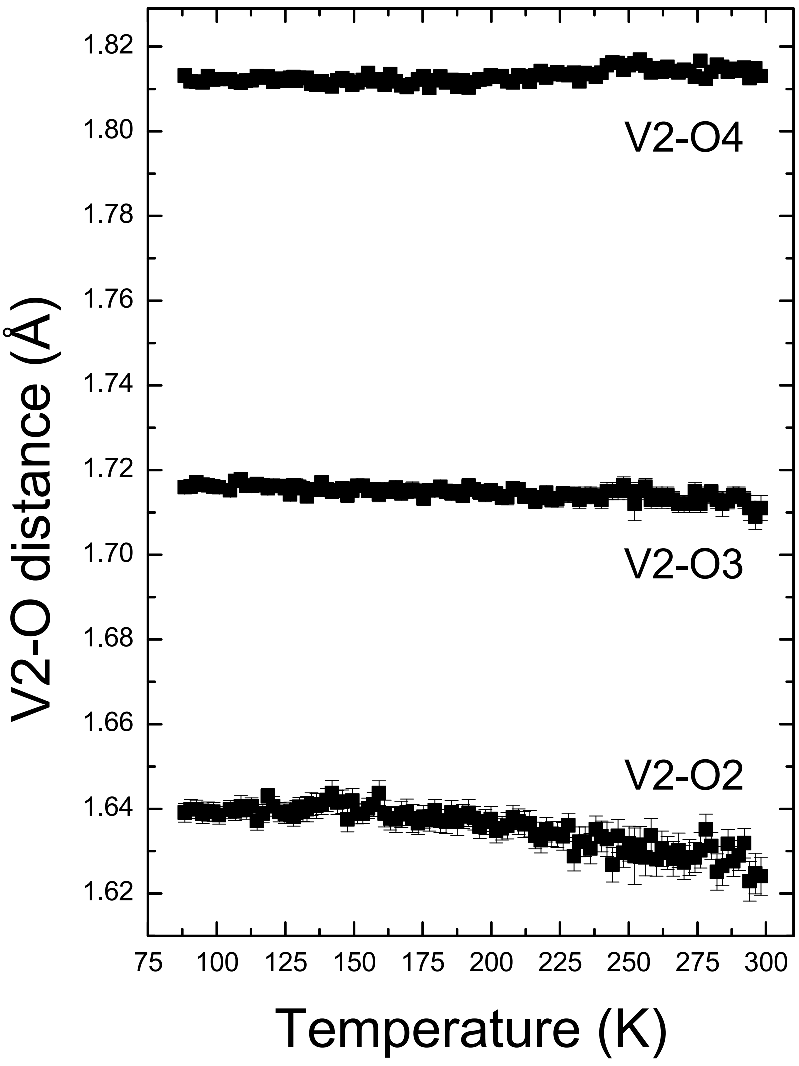

Figure S3 V2-O interatomic distances in the VO_4 tetrahedra as a function of temperature in $K_2V_3O_8$. The standard deviations are drawn when larger than the size of the symbols.

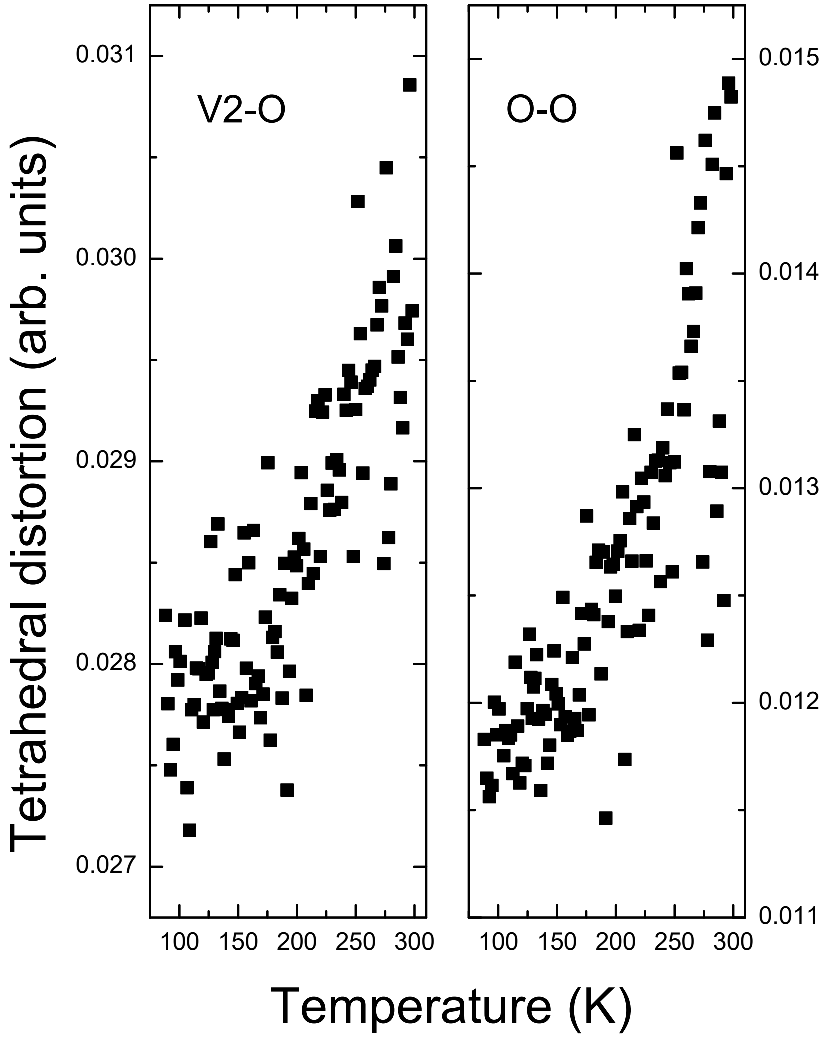

Figure S4 V2-O bond-length and O-O distance tetrahedral distortions in $K_2V_3O_8$.

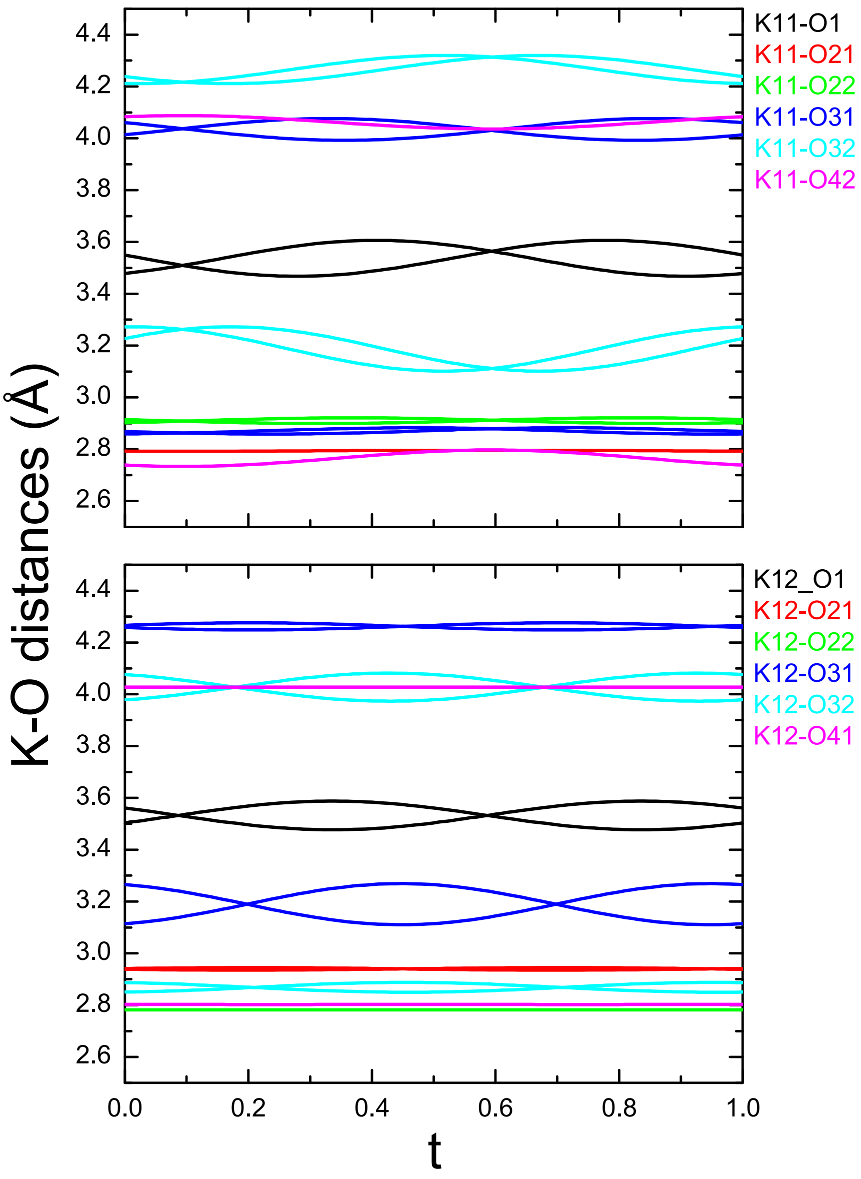

Figure S5 K-O distances in the (3+1)-*d* structure of $K_2V_3O_8$ at 100 K as a function of the internal coordinate *t*.

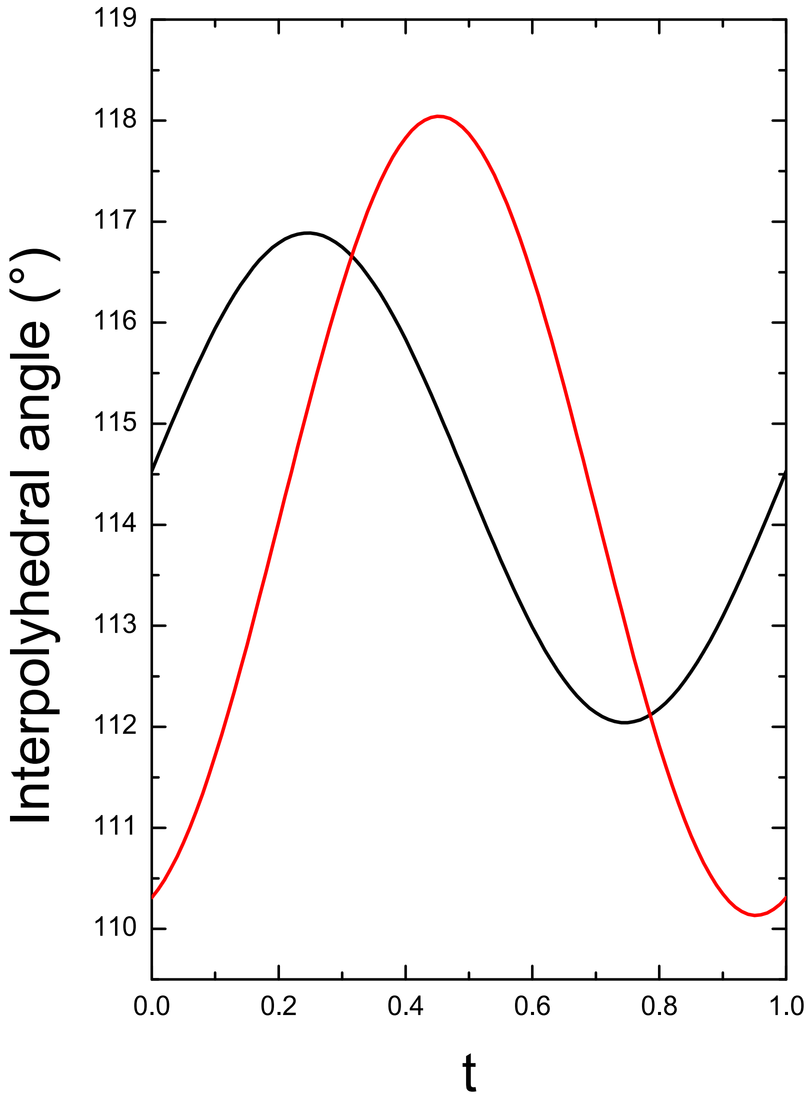

Figure S6 O31-O31-O32 (black line) and O31-O32-O32 (red line) interpolyhedral angles in the (3+1)-*d* model as a function of the internal coordinate *t*.

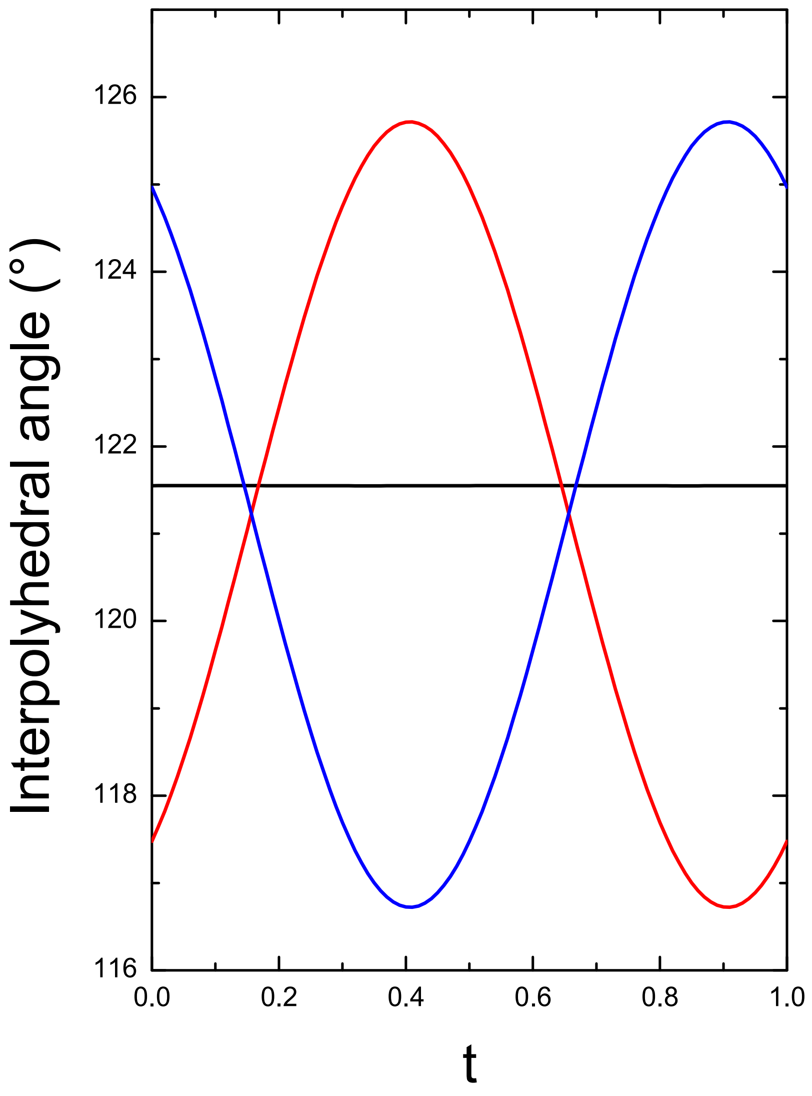

Figure S7 O31-O41-O31 (black line), O32-O42-O32 (red line), and O32-O42-O32 (blue line) interpolyhedral angles in the (3+1)-*d* model as a function of the internal coordinate *t*.
